# Supplementary material for: From sequence to enzyme mechanism using multi-label machine learning
Source: BMC Bioinformatics. 2014 May 19;15:150. doi: 10.1186/1471-2105-15-150 (PMC4229970; doi:10.1186/1471-2105-15-150)
Supplement: Additional file 2 — Java code of ml2db. Additional file ml2db_code.tar.gz contains the Java source code to run the multi-label machine learning experiments and save the results to database. The code’s Javadoc is included. [file 1471-2105-15-150-S2.zip › additional file 2/ml2db/ml2db/doc/mulan/transformations/multiclass/class-use/MultiClassTransformation.html]

Uses of Interface mulan.transformations.multiclass.MultiClassTransformation


JavaScript is disabled on your browser.


- Overview
- Package
- Class
- Use
- Tree
- Deprecated
- Index
- Help

- Prev
- Next

- Frames
- No Frames

- All Classes

## Uses of Interface mulan.transformations.multiclass.MultiClassTransformation

- Packages that use MultiClassTransformation

  | Package | Description |
  | --- | --- |
  |  |  |
  | --- | --- |
  | mulan.classifier.transformation |  |
  | mulan.dimensionalityReduction |  |
  | mulan.transformations.multiclass |  |
- - ### Uses of MultiClassTransformation in mulan.classifier.transformation

    Constructors in mulan.classifier.transformation with parameters of type MultiClassTransformation

    Constructor and Description

    `MultiClassLearner(weka.classifiers.Classifier baseClassifier,
    MultiClassTransformation dt)`

    Initializes learner
  - ### Uses of MultiClassTransformation in mulan.dimensionalityReduction

    Constructors in mulan.dimensionalityReduction with parameters of type MultiClassTransformation

    Constructor and Description

    `MultiClassAttributeEvaluator(weka.attributeSelection.ASEvaluation x,
    MultiClassTransformation dt,
    MultiLabelInstances mlData)`

    Constructor that uses an evaluator on a multi-label dataset using a transformation
  - ### Uses of MultiClassTransformation in mulan.transformations.multiclass

    Classes in mulan.transformations.multiclass that implement MultiClassTransformation

    | Modifier and Type | Class and Description |
    | --- | --- |
    |  |  |
    | --- | --- |
    | `class` | `Copy` Class that implement the Copy transformation method |
    | `class` | `CopyWeight` Class that implement the Copy-Weight transformation method |
    | `class` | `Ignore` Class that implement the Ignore transformation method |
    | `class` | `MultiClassTransformationBase` The base class for multi-class transformation methods. |
    | `class` | `SelectBasedOnFrequency` Class that implement the Select-Max and Select-Min transformation methods. |
    | `class` | `SelectRandom` Class that implement the Select-Random transformation method |

- Overview
- Package
- Class
- Use
- Tree
- Deprecated
- Index
- Help

- Prev
- Next

- Frames
- No Frames

- All Classes
